# Incidence of neuropsychiatric side effects of efavirenz in HIV-positive treatment-naïve patients in public-sector clinics in the Eastern Cape

**DOI:** 10.4102/sajhivmed.v17i1.452

**Published:** 2016-06-30

**Authors:** Razia Gaida, Ilse Truter, Christoffel Grobler

**Affiliations:** 1 Department of Pharmacy, Nelson Mandela Metropolitan University, South Africa; 2 Drug Utilisation Research Unit, Nelson Mandela Metropolitan University, South Africa

## Abstract

**Background:**

It is acknowledged that almost half of patients initiated on efavirenz will experience at least one neuropsychiatric side effect.

**Objectives:**

The aim was to determine the incidence and severity of neuropsychiatric side effects associated with efavirenz use in five public-sector primary healthcare clinics in the Eastern Cape.

**Method:**

The study was a prospective drug utilisation study. A total of 126 medical records were reviewed to obtain the required information. After baseline assessment, follow-up reviews were conducted at 4 weeks, 12 weeks and 24 weeks from 2014 to 2015.

**Results:**

The participant group was 74.60% female (*n* = 94), and the average age was 37.57±10.60 years. There were no neuropsychiatric side effects recorded for any patient. After the full follow-up period, there were a total of 49 non-adherent patients and one patient had demised. A non-adherent patient was defined as a patient who did not return to the clinic for follow-up assessment and medication refills 30 days or more after the appointed date. Some patients (*n* = 11) had sent a third party to the clinic to collect their antiretroviral therapy (ART). The clinic pharmacy would at times dispense a two-month supply of medication resulting in the patient presenting only every two months.

**Conclusion:**

Further pharmacovigilance studies need to be conducted to determine the true incidence of these side effects. Healthcare staff must be encouraged to keep complete records to ensure meaningful patient assessments. Patients being initiated on ART need to personally attend the clinic monthly for at least the first 6 months of treatment. Clinic staff should receive regular training concerning ART, including changes made to guidelines as well as reminders of side effects experienced.

## Introduction

In 2013, an estimated 10% of the South African population was HIV-positive which amounted to 5.26 million people.^1^ This increased to 10.2% in 2014 which amounted to 5.51 million people.^2^ Efavirenz forms part of the first-line treatment of HIV in South Africa and is used as part of the fixed-dose combination (FDC) of triple therapy.

It is acknowledged that almost half of patients initiated on efavirenz will experience at least one neuropsychiatric side effect.^3,4,5^ Commonly reported side effects include dizziness, insomnia, headache, abnormal dreams and impaired concentration.^4,6,7^ These side effects tend to occur within the first few weeks of treatment and spontaneously resolve within that time.^3^ It has also been shown that these side effects may persist longer than a few weeks after the initiation of efavirenz.^5^ They may in fact persist for up to 2 years and this may influence the long-term adherence to efavirenz.^8,9^ The plasma levels of efavirenz as well as genetic polymorphisms in certain populations are thought to be the cause of these neuropsychiatric side effects.^3,10,11^

There have been several studies^7,12,13,14,15^ comparing efavirenz with various other treatments such as nevirapine, protease inhibitors, etravirine and raltegravir. In all instances, efavirenz was seen to induce neuropsychiatric side effects more frequently than these other treatments. The side effects presented most commonly during the first few weeks of treatment and resolved spontaneously after 6 weeks. However, the studies did note that there were instances of late-emergent side effects and recommend that monitoring be an ongoing process.

Efavirenz is effective in reducing viral load in HIV-positive patients. This was shown by Gulick *et al*.^16^ who compared a regimen containing three nucleoside reverse-transcriptase inhibitors (abacavir, lamivudine and zidovudine) with efavirenz-containing regimens. The results of the study showed that efavirenz-containing regimens were virologically superior to those without. Another study^17^ showed that efavirenz treatment resulted in an increase in CD4 cell count and was associated with fewer hypersensitivity reactions and hepatitis than nevirapine. These are some of the reasons for efavirenz being selected as part of the first-line treatment for HIV in South Africa.^7^ However, in light of the problems caused by efavirenz, patient adherence may be affected and treatment interrupted which is undesirable for conditions such as HIV where consistent long-term therapy is required. The South African Guidelines^18^ do acknowledge the neuropsychiatric side effects caused by efavirenz and indicate that previous or current depression or other mental disorders would increase the likelihood of these side effects occurring. The guidelines do not however provide guidance as to how to manage these side effects once they manifest.

Before efavirenz is initiated, patients need to be screened for a history of, or a current mental illness, as such patients may be at a higher risk of developing neuropsychiatric side effects. A South African study conducted in 2008^19^ showed that the prevalence of HIV in people between the ages of 15 and 49 years at the time was 16.9% and that of this population, 43.7% had some kind of mental disorder. The study showed that mild depressive disorder was the most common amongst males (28.1%) and females (30.5%). This was followed by major depressive disorder and alcohol abuse in both sexes.^20^ Another study,^21^ conducted in that same year, included 465 HIV-positive patients and found a mental disorder prevalence of 19%.

The aim of this study was to determine the incidence and severity of neuropsychiatric side effects associated with efavirenz use in HIV-positive patients in a public-sector primary healthcare setting.

## Research design

### Study setting and population

The study was designed as a prospective drug utilisation study. Five public-sector primary healthcare clinics in the Eastern Cape were used as locations for the study. Each of these sites had both an HIV clinic as well as a psychiatric clinic which ensured that patients would be treated at the same facility and not referred.

Patients themselves were not directly involved in the study, and there was no patient contact. Instead, the medical records were reviewed to obtain the required information. Patients were chosen through purposive sampling. The patient files were evaluated to determine whether or not patients met the inclusion criteria. All of the patients who met the outlined criteria were included. Patients who were 18 years or older and diagnosed HIV-positive were included in the study. All of the selected patients were treatment-naïve upon initiation of antiretroviral therapy (ART) and were prescribed efavirenz as part of the triple therapy regimen. Patients with current or previous tuberculosis (TB) infection were also included as were pregnant women. Patients who were previously non-adherent (missed 30 days or more of treatment) and subsequently restarted on ART were excluded. The total sample amounted to 126. For the purposes of this study, the term ‘patient’ will refer to a patient file or medical record. The data capturers at each of the clinics were contacted to obtain access to files. Data capturers are administrative staff at the clinics who electronically capture information concerning HIV-positive patients recorded by nurses. Patients were enrolled between July 2014 and November 2014 and were selected based on the inclusion criteria.

#### Ethical consideration

The study was granted ethics approval by the Nelson Mandela Metropolitan University Research Ethics Committee (Human) (reference number: H14-HEA-PHA-001) as well as the Eastern Cape Department of Health and was conducted according to the principles outlined in the Declaration of Helsinki.^20^ The necessary permissions were obtained from the management of each individual clinic.

### Measurements, outcomes and follow-up procedures

In the public sector of South Africa, HIV medical records are standardised across the country and contain a number of questions to be asked by the healthcare professional to obtain a baseline picture of the patient’s condition. This form was used to obtain the baseline information required for the study. A self-developed data collection tool was used to extract the required information. The information required included age, gender, weight, date of HIV diagnosis, CD4 count at ART initiation, TB infection, pregnancy status, World Health Organization (WHO) stage, whether the patient was a substance abuser and a history of, or current, psychiatric condition. The primary outcome was the incidence and severity of neuropsychiatric side effects associated with efavirenz use as well as the subsequent management of the patient. After the baseline information was obtained, follow-up was conducted at 4, 12 and 24 weeks. In standard practice, patients receive counselling before the HIV test is performed. The healthcare workers explain the significance of a positive result or provide counselling to encourage the continuation of safe practices if the result is negative. For those who test HIV-positive, the patient is counselled to provide an understanding of what measures need to be taken in order to ensure their future well-being. Healthy living, disclosure of status and, if the patient qualifies for ART, the medication and the importance of adherence is discussed with the patient. Once the patient is considered ‘ART-ready’, the patient is initiated on treatment. The time lapse between testing and the initiation of treatment varies between patients. After ART has been initiated, patients present at the clinic on a monthly basis for follow-up assessments and to collect medication. At each visit, the patient would be seen by the wellness clinic nurses for the clinical assessment and the information would be recorded in the file. At the 4-, 12- and 24-week follow-up visits, the file was reviewed by the researcher using the data collection tool.

### Statistical analysis

The data were captured in Microsoft Excel^®^ and analysed using descriptive statistics.

## Results

### Subjects at baseline

A total of 126 patients were followed through the 24-week study period. The participant group was 74.60% female (*n* = 94). The average age was 37.57±10.60 years with females being, on average, younger (35.66±9.83 years) than males (43.19±10.91 years). The difference was seen to be statistically significant (*p* < 0.05). The baseline data are summarised in Table 1.

**TABLE 1:** Baseline data of patients.

Variable	Female†	Male‡
Average age ± standard deviation	35.66±9.83 years	43.19±10.91 years
**Date of diagnosis**		
Before 2008	12	2
2008–2010	5	3
2011–2014	76	27
Not indicated	1	0
**CD4 count**		
> 350 cells/mm^3^	7	3
350–200 cells/mm^3^	29	7
< 200 cells/mm^3^	17	7
Not indicated	41	15
**World Health Organization (WHO) stage**		
Stage 1	46	10
Stage 2	17	6
Stage 3	19	10
Stage 4	4	1
Not indicated	8	5
Pregnant	14	-
**Antiretroviral (ARV) regimen**		
FDC (tenofovir, emtricitabine and efavirenz)	91	31
Zidovudine, lamivudine and efavirenz	1	-
Abacavir, lamivudine and efavirenz	2	1
History of psychiatric illness	1	-
Current psychiatric illness	1	-
Substance abuser	1	-
Alcohol abuser	5	1
Previous TB infection	13	9
Current TB infection	15	6

† *n* = 94;

‡ *n* = 32.

FDC, Fixed-dose combination.

There were 14 (14.89%) female patients who were pregnant at the start of the study, five of whom were in the first trimester, four in the second semester and two in the third trimester. The trimester was not indicated for three patients. All of these women were initiated on efavirenz before the National Department of Health had published an updated guideline^7^ stating that efavirenz could be prescribed to pregnant women regardless of trimester.

Patients were also screened for a history of psychiatric disorders. This was done by means of a single ‘yes’ or ‘no’ question on the standardised medical record asking if the patient had a history of psychiatric illness. One patient had a current symptomatic mental illness and one patient had a history of depression.

Patients were treatment-naïve before the initiation of the efavirenz-containing regimen. The majority of patients (96.83%; *n* = 112) received efavirenz in the form of the FDC tablet. Two patients were not receiving the FDC, one was receiving efavirenz in combination with lamivudine and abacavir, and the other efavirenz in combination with lamivudine and zidovudine. All patients, except one, were receiving efavirenz 600 mg daily. That patient was switched to a lower dose of 400 mg of efavirenz due to weight loss resulting in a total body weight of less than 40 kg. Just over half (53.17%; *n* = 67) of the study population was diagnosed with HIV in 2014. There were 14 patients (11.11%) diagnosed as HIV-positive before 2008 but were initiated on ART only in 2014. This could be due to the CD4 count remaining above the specified threshold of 350 cells/mm^3^ which, according to the South African Guidelines^7^ at that time, was the point at which the patient must be initiated on ART.

The majority of patients (44.40%; *n* = 56) were classified as being in clinical stage 1 of the WHO staging and 23.0% (*n* = 29) being classified as stage 3. Of the patients classified as being stage 3, 62.07% (*n* = 18) were suffering from a concurrent TB infection.

More than half (55.56%) of the patients had a CD4 count of less than 350 cells/mm^3^. This is in keeping with the previous guidelines^22^ which stated that patients with a CD4 count of 350 cells/mm^3^ or less regardless of the clinical stage must be started on ART. The guidelines have since changed to state that patients with a CD4 count of 500 cells/mm^3^ or less must be started on ART regardless of the clinical stage.^18^ There were 10 patients who were initiated on ART with a CD4 count of more than 350 cells/mm^3^. There were three patients in this group with active TB infection at baseline and another three who were pregnant at baseline. Pregnant patients and those with TB must be initiated on life-long ART regardless of the CD4 count.^18^

Substance abuse was not well documented. The standardised medical record includes a ‘yes’ or ‘no’ question querying if the patient is a current ‘drug abuser’. There were 59 (46.83%) patients for whom no response was documented. Alcohol abuse is also in the form of a ‘yes’ or ‘no’ question asking if the patient is a current alcohol abuser and is questioned separately from substance abuse. There were 60 (47.62%) patients for whom no response concerning alcohol abuse was documented. Only one patient admitted to being a substance abuser and six patients admitted to abusing alcohol.

Not many patients were seen to have comorbidities. There were 13 (10.32%) patients each with hypertension and asthma, five (3.97%) patients with diabetes and two (1.59%) patients with epilepsy. Three patients suffered from diabetes and hypertension, one patient suffered from hypertension and asthma, and one patient suffered from hypertension, asthma and epilepsy in addition to HIV.

### Follow-up

There were no neuropsychiatric side effects recorded in the medical records for any patient. One patient reported experiencing a headache; however, this was accompanied by other influenza symptoms and was therefore unlikely to have been caused by efavirenz.

There were non-adherent patients (defined as a patient who did not return to the clinic for follow-up assessment and medication refills 30 days or more after the appointed date) at each follow-up, and this number increased throughout the follow-up period. There were 27 (21.43%), 39 (30.95%) and 49 (38.89%) non-adherent patients after 4, 12 and 24 weeks, respectively. At each follow-up point, there were more female non-adherent patients than male non-adherent patients. After 24 weeks, there were 35 (73.47%; *n* = 49) female non-adherent patients as compared with 14 (28.57%; *n* = 49) male non-adherent patients; however, this was not found to be statistically significant. There were nine patients transferred to different facilities over the follow-up period. Some patients (*n* = 11) sent a third party to the clinic to collect their ART for the month and did not personally present themselves. The clinic pharmacy would also, at times, dispense a 2-month supply of medication resulting in the patient presenting only every 2 months. By the end of the 24-week follow-up, one patient had demised and the cause of death was unknown.

Considering the non-adherent patients more closely (Table 2), six patients were diagnosed with TB co-infection, two were diagnosed as being hypertensive and three were diagnosed as being asthmatic. One patient had both hypertension and asthma. Although patients with comorbidities were prescribed medication for their chronic or infectious diseases, adherence to the medication cannot be guaranteed. Of the 49 non-adherent patients, more than half (56%; *n* = 28) were between the ages of 18 and 35 years. As CD4 cell counts are checked only 12 months after initiation of ART, there were no new results available during the follow-up period.

**TABLE 2:** Characteristics of non-adherent patients.

Variable	Non-adherent patients†
**Male**	14 (28.57%)
Average age±standard deviation	36.86±10.58 years
**Baseline CD4 count**	
> 350 cells/mm^3^	6
350–200 cells/mm^3^	23
< 200 cells/mm^3^	16
Not indicated	25
Pregnant	5
Alcohol abuser	4
Comorbidities	14
TB	6
**WHO stage when recorded non-adherent**	
1	11
2	4
3	8
4	5
Not indicated	21

† *n* = 49.

## Discussion

According to the literature, at least half of the patients who are initiated on efavirenz will experience at least one neuropsychiatric side effect. The clinic records show zero incidence of neuropsychiatric side effects. This could mean that patients were not reporting side effects, clinic staff were not enquiring about side effects or the side effects were not being recorded in the medical records. There were instances of information not being documented in the medical records. This included information such as previous TB infection, whether or not the patient was a substance abuser or whether the patient had a history of a psychiatric disorder.

The questions contained in the standardised medical record for HIV patients were not extensive. A history of psychiatric illness is an important factor in determining the ART regimen, but only a single ‘yes’ or ‘no’ question is devoted to this topic asking the patient if they have a history of psychiatric illness. The patient may not be willing to divulge information concerning a previous mental illness, may not recognise it in themselves or it may not be asked by the healthcare worker. This section should be reviewed, and follow-up questions could be asked in order to determine a history, or current feelings, of depression. It is known that mental illness, particularly depression, serves as a barrier towards adherence.^23^ The ‘yes’ or ‘no’ questions with regard to substance and alcohol abuse may elicit a feeling of shame and the patient may therefore answer in the negative. The South African Guidelines^18^ are not expansive in terms of preparing the patient for ART. It states that the patient must be screened for nutritional status, comorbidities and possible drug interactions and any mental health and substance abuse issues must be addressed.^18^ Further elucidation is not provided concerning the specific questions to be asked or clinical assessments to be done. The way these issues are addressed seems to be at the discretion of individual provinces or facilities. The 2010 HIV Guidelines of Swaziland^24^ dedicate a chapter to mental health and substance abuse. It states that these are underdiagnosed conditions and not properly managed. It recommends that healthcare workers at the primary care level conduct a basic mental health screen and refer patients who appear to have any issues or are at risk of suicide. The Swaziland guidelines^24^ stress that mental illness may affect adherence to ART and is an important issue to consider before initiating treatment. An extensive list of questions is provided to assist healthcare workers in detecting various mental illnesses such as anxiety, depression and suicide risk. In order to provide more comprehensive mental illness screening in South Africa, a similar tool could be adopted.

Some patients did not return to the clinic each month. Patients should be encouraged to personally present themselves at the clinics on a monthly basis for assessment and collection of ART. Third parties should not be allowed to collect medication on behalf of the patient, especially during the first 6 months of therapy when side effects may manifest and this in itself may present barriers to adherence. This also denies the patient any adherence counselling that may be done at these visits. Kranzer *et al*.^25^ found that the rate of non-adherence to ART was highest during the first 6 months of treatment and thereafter declined. It can be understood that the clinics dispense a 2-month supply to patients in order to reduce the patient burden on the dispensary. However, to encourage new patients to come to the clinic, only 1-month supply of medication should be dispensed for at least the first 6 months of treatment in order to ensure retention of the patient.

A study^26^ conducted with patients across South Africa showed an adherence of only 40% after an average of 29 months of treatment. A study focused in the Eastern Cape^27^ showed 37.5% poor compliance to treatment, but no patient discontinued treatment. Another study^28^ set in Cape Town showed only 12.8% non-adherence of a cohort of 242 patients. This shows that adherence varies between provinces in South Africa. It was interesting to note that in this study there were more female non-adherent patients than male non-adherent patients. However, even though the number of female non-adherent patients exceeded the number of male non-adherent patients, the percentage of male non-adherent patients (43.75%) was higher. Kranzer *et al*.^25^ stated that male patients were at higher risk of being non-adherent, whereas female non-adherent patients were more likely to subsequently restart therapy. A retrospective analysis^29^ carried out in South Africa stated that gender was not a predictive factor in terms of non-adherence which shows that there are conflicting ideas surrounding gender as a risk factor for non-adherence to ART. It was also noteworthy that the majority (20.41%; *n* = 10) of patients who were non-adherent by the end of the 24-week follow-up period had initial CD4 counts of below 150 cells/mm^3^.

Studies^30,31^ have been conducted in South Africa to determine the reasons behind non-adherence to ART. Reasons put forth by patients include transport costs, not obtaining time off from work to attend the clinic, transfers between clinics being difficult to complete and resulted in lost paperwork as well as other administration problems.^30,31^ Suggestions have been made to provide patients with vouchers for transport, providing transport to and from the clinic, extending clinic hours for patients who work or providing mobile clinic services in order to decrease the distance patients need to travel to reach the clinic.^30,31^ A study conducted in rural Rwanda^32^ showed that by providing nutritional support, healthcare worker visits to patients’ homes to supervise treatment and a transportation allowance resulted in a 92.3% retention rate. This study showed that high retention rates can be achieved in resource-limited settings.

It was noted that overall the standardised medical record sheet used by the public-sector clinics is not sufficient. The form only allows the most elementary screening of comorbid conditions that would affect the choice of ART prescribed to the patient. Complex conditions such as psychiatric illnesses as well as substance abuse disorders cannot be comprehensively determined by means of single questions. This calls for more integrated healthcare services with psychiatric services performing a more thorough screening of a patient’s mental state. Patients need to be adequately screened to determine those at risk for developing side effects to prevent future hospitalisation. These medical records need to be re-evaluated and a more integrated healthcare service provided to patients.

## Conclusion

Record-keeping at the public-sector clinics is not currently optimal. Healthcare staff must be encouraged to keep complete records in order to ensure meaningful patient assessments with more attention being paid to mental illness, neuropsychiatric side effects, substance abuse and emotional well-being of the patients. Patients being initiated on ART need to personally attend the clinic for monthly assessments at least for the first 6 months of treatment in order to increase the likelihood of retention in care. Suggestions made by other studies to retain patients should be taken into consideration. These included home visits by healthcare workers, extended clinic hours and mobile clinics as well as providing patients with transport or remuneration for transport to attend the clinics. The medical information sheet used to obtain the patient’s medical history should be re-examined and improvements made where necessary. Clinic staff should receive regular training concerning ART including changes made to guidelines as well as reminders of side effects experienced and suggestions for questions to be asked when the patient attends follow-up sessions.

### Limitations

The small sample size serves as a limitation to the study as well as the fact that the patients were contained in a single district within the province. The inclusion of patients stretching across a broader location would be desirable. The record-keeping at these facilities has not proven optimal, and improvement in this regard would serve to improve future studies.
